# Development of Tooth Brushing Recommendations Through Professional Consensus

**DOI:** 10.1016/j.identj.2023.10.018

**Published:** 2023-12-05

**Authors:** Anne-Marie Glenny, Tanya Walsh, Makiko Iwasaki, Elham Kateeb, Mariana Minatel Braga, Philip Riley, Paulo Melo

**Affiliations:** a Division of Dentistry, School of Medical Sciences, University of Manchester, UK; b Iwasaki Dental Clinic, Kyoto, Japan; c Oral Health Research and Promotion Unit, Al-Quds University, Jerusalem, State of Palestine; d School of Dentistry, University of São Paulo, Brazil; e Faculty of Dental Medicine, Laboratory for Integrative and Translational Research in Population Health (ITR), University of Porto, Portugal

**Keywords:** Tooth brushing, Oral hygiene, Dental devices, Home care, Mouth rinse, Interdental cleaning

## Abstract

**Introduction:**

Despite being a largely preventable disease, untreated caries of permanent teeth is estimated to affect almost 2 billion people worldwide, which is followed by severe periodontal disease. The aim of this work was to provide a professional consensus on tooth brushing methods and associated oral hygiene behaviours and develop evidence-informed recommendations.

**Methods:**

An initial scoping search was undertaken to identify systematic reviews of relevance and key questions. This was followed by comprehensive evidence mapping of the literature focussing on systematic reviews and clinical guidelines. Electronic searches of several databases including MEDLINE (via Ovid), Embase (via Ovid), Epistemonikos, and The Cochrane Library were undertaken from 2000 to May 2022, alongside a guideline repository search. Considered Judgement Forms were developed detailing the underpinning evidence, balance between benefits and harms, potential impact on the population, and feasibility of implementation. An online survey comprising 22 draft recommendations was distributed to international members of all FDI committees, including the FDI Council. Participants were asked to indicate to what level they agreed or disagreed with for each recommendation and to provide feedback. The Considered Judgement Forms were provided for reference.

**Results:**

Three hundred ten records were identified and mapped to different aspects of tooth brushing methods and associated behaviours. Research literature informed 7 Considered Judgement Forms comprising 12 questions with draft recommendations. Twenty-five participants from Asia, Europe, North and South America, Africa, and Australia provided feedback on the recommendations. More than 70% of respondents showed agreement with 21 of the 22 draft recommendations. Final recommendations were drafted with associated strength of recommendation.

**Conclusion:**

Using a robust methodology and an international professional consensus, a set of evidence-informed recommendations was developed. These recommendations provide clinicians with practical guidance to facilitate communications with patients that may help to reinforce individual-level preventive strategies.

## Introduction

The global burden of oral diseases represents a major public health concern. Using data from the Institute of Health Metrics and Evaluation global burden of disease model for oral diseases, the World Health Organization reports that around 3.5 billion people were affected by oral diseases in 2019. Untreated caries of permanent teeth was the most prevalent oral disorder, affecting almost 2 billion people worldwide, followed by severe periodontal disease, untreated caries of the primary teeth, and edentulism.^1^^,^^2^ Further, inequalities in oral health persist, with the greatest negative impacts borne by those most disadvantaged.^1^

The importance of attaining and maintaining good oral health cannot be overestimated. In addition to pain and infection, the consequences of poor oral health can include difficulties in eating and sleeping and time off work or school to attend appointments for dental treatment. Whilst daily tooth brushing with fluoride-containing toothpaste is encouraged, to our knowledge, there is no current professional consensus on recommendations for tooth brushing and associated oral hygiene behaviours for the general population or individuals with additional health care needs. Resources produced by the FDI World Dental Federation (FDI), national dental associations, and educational bodies suggest a variety of tooth brushing techniques and behaviours (eg, frequency and duration of tooth brushing) with guidance varying internationally.^3^

Comprising 200 national dental associations and specialist groups in more than 130 countries, the FDI serves as the principal representative body for more than 1 million dentists worldwide.^4^ In accordance with the organisation's vision to lead the world to optimal oral health, the FDI commissioned a Task Team to develop professional consensus recommendations on tooth brushing methods and associated behaviours for the general population of children, adults, and the elderly based on published evidence, clinical guidelines, and expert professional consensus. Additional recommendations were to be considered for specific subpopulations. These recommendations would subsequently be used to develop visual and chairside guides to facilitate dissemination of the recommendations to patients and clinicians.

## Methods

### Topic proposal and selection

The Task Team developed a provisional scope for the guidance document for children, adults, and the elderly considering factors such as:

- Toothbrush characteristics: powered or manual, type and shape of the bristles, area, and hardness of the active part of the toothbrush
- Duration, timing, and frequency of tooth brushing
- Whether to spit or rinse before or after tooth brushing
- Type of toothpaste, including degree of abrasiveness of toothpaste appropriate for the specific brush type
- Interdental cleaning

The guidance could also include brushing method for specific subpopulations such as autonomous patients with any of physical, cognitive, and motor disabilities or their caregivers or for specific clinical contexts such as postsurgery, periodontal treatment, dentin sensitivity, acute necrotising ulcerative gingivitis, cleft palate, and so on.

### Scoping search

An initial scoping search was undertaken to indicate the volume of relevant literature and any existing evidence gaps and to refine the scope and clinical questions to be addressed. Identified systematic reviews were then mapped to specific questions.

### Agreement on scope and key questions

Following the scoping search the Task Team, in conjunction with FDI representatives, determined the final scope of the guidance document and key questions. The effectiveness of different tooth brushing techniques (eg, Bass technique, Fone technique) was not prioritised as a key question due to the lack of consistency in the definition of tooth brushing techniques in the literature.

### Evidence mapping

Comprehensive evidence mapping of the literature focussing on existing systematic reviews and guideline documents was then undertaken. Electronic searches of the following databases were undertaken in May 2022: MEDLINE via Ovid, Embase via Ovid, Epistemonikos, and The Cochrane Library (including CDSR and DARE), in conjunction with an electronic search of guideline repositories. Searches focused on contemporary underpinning evidence and were limited to 2000 onwards with no restriction on language of publication (see Appendix A). A wide-ranging search strategy was constructed to cover tooth brushing/cleaning and oral health in general. More focussed searches were undertaken for specific issues such as storage and replacement of brushes.

Single-episode brushing studies, in vitro studies, reviews that had been subsequently updated, and commentaries were all excluded. Additional manual searching of guideline repositories and follow-up of citations from included studies was undertaken.

Following the searches, all identified records were imported to EndNote X9 for screening independently by 2 authors. If eligibility was unclear from the abstract alone, then the full text was obtained. Other relevant sources of evidence identified by the authors at any point in the guidance development process were also considered.

### Data extraction and appraisal

A single author extracted data from the included systematic reviews, guidance documents, and primary studies using a predefined Evidence Summary Form. All data extraction and evidence summary tables were double-checked by a second author.

### GRADE assessment

The Grading of Recommendations, Assessment, Development, and Evaluation (GRADE) approach was used to summarise the overall certainty of evidence presented by each included systematic review or guideline.^5^ Where no GRADE assessment was reported, the publication was assessed according to criteria specified by GRADE (study limitations [risk of bias], inconsistency of results, indirectness of evidence, imprecision, and publication bias) and assessed at 1 of 4 levels, as indicated in Table 1.^6^ A subset of the GRADE assessments was double-checked by a second reviewer.

**Table 1:** GRADE levels of certainty for a body of evidence for a given outcome.

• **High certainty:** We are very confident the true effect lies close to that of the estimate of the effect. • **Moderate certainty:** We are moderately confident in the effect estimate; the true effect is likely to be close to the estimate of the effect, but there is a possibility that it is substantially different. • **Low certainty:** Our confidence in the effect estimate is limited; the true effect may be substantially different from the estimate of the effect. • **Very low certainty:** We have very little confidence in the effect estimate; the true effect is likely to be substantially different from the estimate of effect.

### Development of recommendations

The Evidence Summary Forms were used to draft Considered Judgement Forms (CJFs), providing a summary of the identified evidence for each key question. These forms detailed the certainty of the evidence, the balance between benefits and harms, the potential impact on the population, the feasibility of implementation, and a draft provisional recommendation.

Authors reviewed the Evidence Summary Forms and CJFs and provided feedback on the provisional strength of recommendations through an informal discussion to achieve consensus (Table 2). All recommendations were linked to the supporting evidence.

**Table 2:** Strength of recommendations.

**Strong**: The Task Team is confident the desirable effects of an intervention outweigh its undesirable effects *or* that the undesirable effects outweigh desirable effects.^6^
**Conditional**: The desirable effects probably outweigh the undesirable effects *or* undesirable effects probably outweigh the desirable effects, but appreciable uncertainty exists.^6^
**Good practice**: Clinical opinion suggests this advice is well established or supported; no robust underpinning research evidence exists. Good practice points may be based on extrapolation from research on related topics and/or clinical consensus, expert opinion, and precedent.

### Achieving consensus

A modified Delphi approach was undertaken for the consensus process. An online survey was constructed and distributed to members of all 5 FDI standing committees and the FDI Council. For each draft recommendation, participants were asked to indicate the level to which they agreed/disagreed with each recommendation using a Likert scale ranging from 1 (strongly disagree) to 7 (strongly agree) and to provide comments. Agreement was classed as a response from 5 to 7 on the Likert scale; consensus on a recommendation was reached when ≥70% of participants agreed with the recommendation. The CJFs were provided for reference, outlining the underpinning research evidence where available; “good practice” recommendations were drafted in the absence of any underpinning research literature. This process was to be repeated for a maximum of 3 rounds or until consensus was reached if sooner.

## Results

### Scoping search, agreement on scope, and key questions

A total of 310 records were identified and mapped to different aspects of tooth brushing in accordance with the topic proposals.

### Evidence mapping

Evidence Summary Forms were developed for the key questions, indicating their suitability for general and specific populations.

### Considered judgements and draft recommendations

Seven CJFs outlining the underpinning evidence were constructed, comprising 12 key questions agreed by the Task Team, with 22 recommendations (see Supplementary File).

The focus of the CJFs and the key questions are listed in Table 1, with references to the underpinning evidence. There were 4 additional key questions where there was felt to be uncertainty about the effects of an intervention, with insufficient evidence to support a decision:

• “How effective are miswak/wooden sticks?” (CJF4). There is insufficient evidence to determine the effectiveness of different types of wooden sticks/chewing sticks. The benefits and harms could not be determined from the available evidence.
• “What is the best type of toothpaste (xylitol, arginine, herbal)?” (CJF5). There is limited or low/very low certainty evidence for the addition of xylitol to fluoride toothpaste, the effect of herbal toothpastes, or the addition of arginine to fluoride toothpaste for preventing caries.
• “Is there a difference between fluoride formulations (eg, stannous fluoride [SnF2] vs sodium monofluorophosphate [SMFP] vs sodium fluoride [NaF]) in the caries preventive effect of toothpastes and, if so, are these clinically significant?” (CJF6). There is limited or low/very low certainty evidence for the effect of different fluoride formulations.
• “What is the optimum amount of fluoride toothpaste to use for daily tooth brushing?” (CJF6). There is limited or low/very low certainty evidence for the optimum amount of fluoride toothpaste for preventing caries.

### Achieving consensus

Twenty-five participants provided feedback on the draft recommendations, for a response rate of 49%. Most participants were from Europe (9 participants) and Asia (8 participants), followed by North or South America (5 participants), Australia/Oceania (1 participants), and Africa (1 participant). We were unable to obtain location from 1 participant.

More than 70% of participants expressed agreement with 21 of the 22 draft recommendations. All participants agreed on 3 draft recommendations: “Parents or carers should continue to assist or supervise brushing until a child can brush their own teeth effectively,” “Patients should be advised to regularly clean their teeth and gums using either a manual or rechargeable powered toothbrush,” and “Patients with physical disabilities may benefit from grip handles or other adaptations (eg, 3-sided toothbrushes, powered toothbrushes).” The item that received the greatest variability in response was “Where a person can't spit, wiping their mouth with a cloth after brushing may help,” with only 48% of participants agreeing with this statement. After reviewing feedback, this draft recommendation was subsequently changed to “Where a person is unable to spit, use an appropriate amount of toothpaste and assist with clearing excess from around their mouth after brushing.” The statement “Patients should be advised to regularly clean their teeth and gums, using either a manual or rechargeable powered toothbrush” was qualified with “The effectiveness of both manual and powered toothbrushes is influenced by user technique.”

Feedback provided by the participants resulted in minor changes to the wording of the draft recommendations.

As the percentage agreement for all but one of the recommendations exceeded the threshold of 70%, no further online rounds were deemed necessary. Based on the supporting evidence, the Task Team provided the accompanying strength of recommendations following informal discussion.

The focus of the Considered Judgement Forms and the key questions are listed in Table 3, with references to the underpinning evidence. There were 4 additional key questions where there was felt to be uncertainty about the effects of an intervention, with insufficient evidence to support a decision.

**Table 3:** Final recommendations and strength of recommendations.

Considered Judgement Form (CJF) and underpinning evidence	Question	Final recommendation	% Agreement	Strength of recommendation
CJF1 What is the best timing, frequency, and duration for tooth brushing?^7^^,^^8^^,^^9^^,^^10^^,^^11^^,^^12^^,^^13^^,^^14^^,^^15^^,^^16^	When should you brush your teeth?*	For adults and children, teeth should be brushed the last thing at night or before bedtime and at least one other time.	96%	STRONG^a^
	How often should you brush your teeth?*	It is recommended for adults and children that tooth brushing should be undertaken/supported at least twice a day.	96%	STRONG^a^
	How long should you brush your teeth for?*	For adults and children, teeth should be brushed for long enough to ensure that all tooth surfaces are cleaned effectively. This may take around 2 minutes, depending upon the number of teeth present and manual dexterity.	96%	STRONG^a^
CJF2 Tooth brushing in children: When should it start and for how long should it be supervised?^9^^,^^12^^,^^14^^,^^15^^,^^17^^,^^18^^,^^19^^,^^20^	When should children start having their teeth brushed?	Parents or carers should commence brushing/cleaning their children's teeth with either a soft toothbrush or cloth as soon as the first tooth erupts. If using a cloth, parents or carers should change to using a toothbrush when the child's molars erupt.	92%	GOOD PRACTICE
	Until what age should children be supervised when brushing their teeth?	Parents or carers should continue to assist or supervise tooth brushing until a child can brush their own teeth effectively.	100%	STRONG^a^
CJF3 Should you rinse or spit before/after tooth brushing?^9^^,^^21^^,^^22^	Should you rinse or spit before/after tooth brushing?*	To maintain fluoride concentration levels in the mouth, spit out excess toothpaste after brushing. Rinsing with water after brushing is not recommended. If a mouth rinse is used, then this should be at a time other than immediately after tooth brushing.	80%	GOOD PRACTICE
		Young children may need close supervision to ensure excess toothpaste is spat out after brushing.	84%	GOOD PRACTICE
		Where manual dexterity may limit brushing technique, rinsing the mouth with water prior to brushing may help dislodge food debris.	72%	GOOD PRACTICE
		Where a person is unable to spit, use an appropriate amount of toothpaste and assist with clearing any excess from around their mouth after brushing.	48%	GOOD PRACTICE
CJF4 Toothbrush type^9^^,^^23^^,^^24^^,^^25^^,^^26^^,^^27^^,^^28^^,^^29^^,^^30^^,^^31^^,^^32^^,^^33^^,^^34^^,^^35^	What is the most effective type of toothbrush for maintaining oral health (powered or manual)?	Patients should be advised to regularly clean their teeth and gums using either a manual or rechargeable powered toothbrush. The effectiveness of both manual and powered toothbrushes is influenced by user technique.	100%	STRONG^b^
	What is the best toothbrush head type/shape?	Patients should be advised to use a small toothbrush head with bristles of soft or medium texture, taking into consideration the variation in texture across manufacturers.	84%	CONDITIONAL^c^
		Patients with physical disabilities may benefit from grip handles or other adaptations (eg, 3-sided toothbrushes, powered toothbrushes)	100%	CONDITIONAL^d^
CJF5 How effective are interdental cleaning devices for preventing and controlling periodontal diseases and preventing caries?^9^^,^^36^^,^^37^^,^^38^^,^^39^^,^^40^^,^^41^^,^^42^	How effective are interdental cleaning devices for preventing and controlling periodontal diseases and preventing caries?	The choice of interdental cleaning approach may depend on spacing of teeth. Interdental brushes, single-tufted brushes, and dental floss are all options for cleaning and should be selected based on interproximal size and effectiveness within the space following professional advice.	96%	CONDITIONAL^e^
CJF6 What is the most effective type of toothpaste for maintaining oral health?^9^^,^^43^^,^^44^^,^^45^^,^^46^^,^^47^^,^^48^^,^^49^^,^^50^^,^^51^^,^^52^^,^^53^	What concentration of fluoride toothpaste is most effective?	Children should use fluoridated toothpaste containing at least 1000 ppm fluoride.	84%	STRONG^f^
		For those aged 7 years or older use fluoridated toothpaste containing 1000 to 1500 ppm fluoride.	92%	STRONG^f^
		For those with active caries or who are susceptible to dental caries, the prescription and use of a higher fluoride concentration toothpaste (2800 ppm or 5000 ppm according to manufacturers’ guidance) as part of an overall prevention strategy should be considered (for adults and children older than 10 years old). This should be reviewed at routine oral health assessments.	88%	CONDITIONAL^g^
		For those aged 10 years or older and undergoing orthodontic treatment with a fixed appliance, consider the prescription of a high-fluoride toothpaste throughout the treatment phase.	88%	CONDITIONAL^h^
		Where sensory sensitivities are a concern, mild flavoured toothpastes may be preferred. For certain groups including those with sensory sensitivities, vulnerable airways, dysphagia, and cognitive impairment, low-foaming toothpastes may be preferred (eg, sodium lauryl sulphate free).	88%	GOOD PRACTICE
CJF7 How should a toothbrush be stored? How often should a toothbrush be replaced?^13^^,^^54^^,^^55^^,^^56^^,^^57^	How should a toothbrush be stored?	The toothbrush should be stored in an upright position after use and allowed to air-dry.	88%	GOOD PRACTICE
		The toothbrush should be rinsed thoroughly after each use to remove any remaining paste and debris.	96%	GOOD PRACTICE
		Toothbrushes should not be shared between family members.	96%	GOOD PRACTICE
	How often should a toothbrush be replaced?	Toothbrushes should be replaced every 3 to 4 months or more often if the bristles are visibly matted or frayed and immediately after an infection/disease.	96%	GOOD PRACTICE

⁎ No systematic review evidence to answer this question.

a Whilst there is an absence of underpinning evidence for these recommendations, the Task Team is highly confident that desirable consequences outweigh undesirable or undesirable consequences outweigh desirable.

b Evidence from a well-conducted systematic review (moderate certainty) comparing powered and manual toothbrushes indicating that powered toothbrushes may be more effective in reducing gingival index/plaque scores; the clinical importance of the benefit is unclear.

c Evidence from 2 systematic reviews (low certainty) for bristle type; one review suggested that better results for plaque and gingivitis may be achieved from medium or hard toothbrushes whilst a second review showed that soft and extra-soft toothbrushes tend to be safer, with fewer gingival lesions. There is insufficient evidence regarding size of toothbrush head.

d Based on low-/moderate-certainty evidence of no benefit of powered toothbrushes in comparison to manual brushes; recommendation based on potential ease of use. There is limited evidence to suggest the use of a triple-headed manual toothbrush instead of a single-headed manual toothbrush with respect to plaque removal for care-dependent individuals.

e Low-certainty evidence that interdental dental cleaning devices, as adjuncts to tooth brushing, remove more dental plaque and reduce gingival inflammation than brushing alone; the clinical importance of the findings is unclear. There is no evidence to determine whether dental cleaning devices reduce caries when compared to tooth brushing alone.

f Fluoride toothpaste of 1000 ppm F or higher prevents caries in both the permanent and primary dentition (moderate/high certainty). The greatest caries preventive effect was observed for 1500 ppm F compared with 0 ppm F.

g Evidence from a dose-response analysis in a well-conducted systematic review. There is an absence of direct evidence from primary studies. Evidence for the effects of 5000 ppm F concentration toothpaste comes from studies of root caries.

h The use of a high-fluoride toothpaste throughout orthodontic treatment might be more effective than a conventional fluoride toothpaste for preventing early tooth decay (low certainty).

## Discussion

Evidence-informed recommendations were developed through professional consensus to provide guidance on tooth brushing methods and associated behaviours, including toothbrush and toothpaste type, interdental cleaning, and storage and replacement of toothbrushes. Additional recommendations for populations with specific needs have been provided where available and as appropriate. However, individualised oral hygiene advice may additionally be required to optimise oral hygiene behaviours in specific subgroups such as individuals with periodontitis. These recommendations have been taken forward in FDI visual and chairside guides for the purposes of dissemination to national dental associations, oral health professionals, and patients.

There was high agreement from the professional panel for many of the key questions, particularly for the timing, frequency, duration, and supervision of tooth brushing. All but one participant agreed with the recommendation that teeth should be brushed last thing at night or before bedtime. Although there is no systematic review evidence to inform this recommendation, there is evidence to indicate that brushing at night and not eating thereafter will enable fluoride protection during the night and avoid the retention of carbohydrates in the mouth during sleep time when the salivary flow is low.^58^ Eating after tooth brushing raises the risk of having a sustained demineralisation process during sleep.^58^^,^^59^

There was similarly high agreement that tooth brushing should be carried out at least twice daily, before bedtime and on at least one other occasion, in order to remove plaque and to maintain a favourable level of fluoride during the daytime.^16^ Maintaining levels of fluoride in the mouth throughout the day and night has been shown to play a major part in caries prevention.^50^^,^^60^ In high–caries risk populations, additional tooth brushing may be indicated, especially in countries that do not add fluoride to the water supply and where the sole source of fluoride exposure is through brushing with a fluoridated toothpaste.

Regarding the duration of brushing, teeth should be brushed for long enough to ensure that all surfaces are cleaned effectively. Ensuring that all surfaces are cleaned effectively was believed to be more important than adhering to a specific duration of brushing. Further, the effectiveness of different tooth brushing techniques was not prioritised as a key question due to the lack of consistency in definition of tooth brushing techniques in the literature. The recommendations state that the effectiveness of manual and powered toothbrushes is influenced by user technique, and so dental professionals should ensure that patients are taught to brush comprehensively using, for example, (modified) Bass or Fones technique, and cleaning all surfaces and gums. For people with periodontitis, particular attention should be given to the gumline.

All participants agreed that tooth brushing should be supervised by a parent or carer until a child is able to brush their own teeth effectively, thereby creating a lasting competency in tooth brushing behaviour.^61^^,^^62^^,^^63^ The ability to brush effectively will differ from one child to another, and so the appropriate age to stop supervised tooth brushing should be determined by the child's parent or carer. There was some variation in responses to the recommendation that parents or carers should commence brushing or cleaning their child's teeth with either a soft toothbrush or cloth as soon as the first tooth erupts. From participant feedback, the use of a cleaning cloth was the source of the variation, but there was a clear understanding that teeth should be brushed/cleaned as soon the first teeth erupt. While this alternative method of teeth cleaning can be useful in some populations, cleaning with a soft toothbrush should be prioritised over cleaning with a cloth wherever possible.

There was greater variation in responses for recommendations in other areas such as the use of interdental cleaning devices. The scoping search identified many published systematic reviews evaluating the effects of interdental brushing and flossing, of varying quality. All are based on trials of unclear or high risk of bias and focus on the reporting of plaque and/or gingivitis. The effectiveness of interdental cleaning may be influenced by user technique. Further, interdental cleaning may not be routine for many people, and children and adolescents may neglect daily flossing.^64^^,^^65^ The lack of consensus in the literature regarding the most effective device was reflected in the responses of the professional panel. The device should be selected based on interproximal size and effectiveness within the space following professional advice. With so many options and clinical indications, patients should be guided by the knowledge and experience of oral health professionals.

Rinsing after tooth brushing was another area of variation, with some participants suggesting rinsing with a minimal amount of water or mouth rinse, as indicated in their local guidance. Rinsing with water or a mouth rinse after brushing can have the undesirable effect of removing all traces of fluoride toothpaste from the oral cavity. However, rinsing with a mouthwash at a time other than when tooth brushing may be beneficial in, for example, high-risk patients and rinsing with water before tooth brushing may confer a benefit for those who have difficulties with manual dexterity and brushing technique.^66^^,^^67^

Fluoride concentration provoked discussion amongst the participants and members of the Task Team, reflecting the international variability in clinical guidance and product availability (not all fluoride concentrations are readily available; for example, in Japan the maximum fluoride concentration authorised is 1500 ppm and high-concentration fluoride toothpaste such as 5000 ppm is not yet permitted as a commercial product), access to care, and availability of other sources of fluoride through school- or community-based caries prevention programmes or water fluoridation schemes. Overexposure to fluoride may also be a concern in some populations and in localities where fluoride is added to the water supply. However, real-world data from Brazil, for example, have shown that fluorosis levels tend to be mild even in populations where a fluoridated water supply is available and fluoride toothpaste is strongly advocated.

Several recommendations raise financial considerations. For instance, the use of toothpastes with different fluoride concentrations from infant through to adolescent may be recommended, but the cost of purchasing several toothpastes of different concentrations for different family members may be prohibitive. For some families, it may be more appropriate to focus on using a smaller amount of a family toothpaste rather than purchasing several different products. In addition, the total soluble fluoride availability in relation to toothpaste concentration should be considered.^68^ Similarly, whilst the evidence suggests that powered toothbrushes are more effective than manual toothbrushes in reducing plaque and gingivitis, the initial and ongoing costs may be unaffordable to many, possibly increasing oral health disparities.

### Strengths

Transparency of process and inclusivity were key strengths of this work. The scope and remit of the work was dictated by international members of all 5 FDI standing committees and the FDI Council. The Task Team comprised clinicians and methodologists, thus ensuring clinical expertise and a rigorous methodological approach. International recruitment to the Task Team and online panel ensured that the views of professionals with different backgrounds and theoretical/practical preferences, operating under different health care systems and clinical settings, were represented.

### Limitations

The electronic searches were limited to articles published from 2000 onwards to develop recommendations based on contemporary evidence. This restriction on publication date was a pragmatic decision, and the Task Team felt that the development of the recommendations would not be negatively impacted by this restriction.

Some of the recommendations are open to interpretation. Underpinning evidence was not available for all key questions, and therefore it was not possible to be wholly directive in all recommendations. There is therefore potential for deviations in behaviour from that intended when implementing the recommendations. However, the remit of the Task Team was to develop professional consensus recommendations to guide and support clinical practice to be used in conjunction with expert clinical judgement, taking into account individual presentation and clinical setting.

In summary, 22 recommendations for tooth brushing and associated behaviours were developed through professional consensus using a modified Delphi process with participants from different backgrounds, clinical specialties, and health care systems. These recommendations provide practising clinicians with practical guidance in tooth brushing methods and behaviours to facilitate communications with their patients and may help to reinforce individual-level preventive strategies. The accompanying visual and chairside guides will be informative in disseminating the professional consensus recommendations.

## Conflict of interest

None disclosed.

## Appendix A

Electronic search strategy to identify systematic reviews.

Medline via OVID

1 exp Oral hygiene/ 20823

2 Oral health/ 20128

3 exp dental caries/ 50113

4 (caries or carious).ti,ab. 46035

5 exp Periodontal diseases/ 95163

6 (periodont$ or gingiv$).ti,ab. 105883

7 ((oral or dental) adj2 (hygiene or care)).ti,ab. 34382

8 ((mouth or teeth) adj3 care).ti,ab. 659

9 (plaque$ adj5 (remov$ or control$)).ti,ab. 6133

10 Dental plaque/ 17363

11 1 or 2 or 3 or 4 or 5 or 6 or 7 or 8 or 9 or 10 231805

12 (toothbrush$ or tooth brush$ or floss$ or "chewing stick$" or miswak or "wood stick$" or toothpick$).ti,ab. 8804

13 ((dental or oral or mouth or interdental or interproximal or tooth or teeth or orthodontic or brace$ or bracket$) adj3 (irrigat$ or clean$ or brush$ or aid$)).ti,ab. 9236

14 12 or 13 14707

## Appendix B Supplementary materials

Image, application 1
