# Supplementary material for: Development of Tooth Brushing Recommendations Through Professional Consensus
Source: Int Dent J. 2023 Dec 5;74(3):526–35. doi: 10.1016/j.identj.2023.10.018 (PMC11123540; doi:10.1016/j.identj.2023.10.018)
Supplement: Supplementary file 1 [file mmc1.docx]

**Supplementary File 1**

The completed Considered Judgement Forms outlining the underpinning evidence to support the draft recommendations are provided in Supplementary File 1, Tables CJF1 to CJF7.

| CJF1 | Considered Judgement Form |
| --- | --- |
| **Overarching Question**  **What best timing, frequency, and duration for toothbrushing?**   - *When should you brush your teeth?* - *How often should you brush your teeth?* - *How long should you brush your teeth for?* | |
| **1. Summary of evidence** There is no high certainty evidence to determine the optimal timing, frequency, and duration of toothbrushing for the prevention of caries.  Multiple clinical guidelines have highlighted the lack of evidence regarding the timing of toothbrushing, with all guidelines recommending toothbrushing last thing at night/before bed (1, 2, 3). For both adults and children there are also recommendations that toothbrushing should take place on at least one other occasion (1, 3). It should be noted that the SIGN 2014 guidelines form the basis for several of the recommendations in other guideline documents. One guideline document, aimed at adults requiring assistance, suggests that toothbrushing should ideally occur after every meal (2). There is no evidence to support this suggestion.  Most identified guidelines, making recommendations on toothbrushing frequency, recommend twice daily brushing, or ‘at least’ twice daily (1, 3, 4, 5, 6, 7, 8, 9). Those that provide evidence to support this recommendation reference a systematic review that shows a 14% increase in prevented fraction (95% CI 6% to 22%) with twice daily brushing with a fluoride toothpaste compared to once daily brushing with a fluoride toothpaste (10). There is no high certainty evidence to determine the impact of toothbrushing frequency itself. However, there is low certainty evidence that toothbrushing with fluoride toothpaste should take place at least twice daily.  There is insufficient evidence to specify a specific duration of brushing; clinical guidelines suggest at least 2 minutes (2, 3, 7, 8, 9). In addition, SDCEP (2018) suggest that, although professionals often advise brushing for two minutes, the main point is to ensure that sufficient time is taken for all tooth surfaces to be cleaned effectively (8). | |
| **2. Quality and quantity of evidence**  There is no high certainty evidence regarding the optimal timing, frequency, and duration of toothbrushing for the prevention of caries. Recommendations are considered ‘best practice’. | |
| **3. Subgroup considerations**  Children: supervision (covered in “*Until what age should children be supervised when brushing their teeth?”)* | |
| **4. Consistency**  Current clinical guidelines are reasonably consistent in terms of the recommendations made | |
| **5. Balance of effects**  *To be considered* | |
| **6. Generalisability and applicability**  There are no major concerns regarding the generalisability or applicability of the recommendations presented in the identified guidelines. | |
| **7. Values and preferences**  *-* | |
| **8. Acceptability**  *-* | |
| **9. Feasibility**  *-* | |
| **10. Other factors**  *-* | |

| CJF2 | Considered Judgement for Recommendations |
| --- | --- |
| **Overarching Question**  **Toothbrushing in children: when should it start and for how long should it be supervised?**   - *When should children start having their teeth brushed?* - *Until what age should children be supervised when brushing their teeth?* | |
| **1. Summary of evidence** There is no high certainty evidence to determine the optimal age at which children should start having their teeth brushed. Multiple guidelines recommend that toothbrushing should commence as soon as the first tooth erupts (1, 3, 8, 9), or no later than the eruption of the first tooth (11).  Where guidelines provide evidence to support this recommendation it comes from observational study data (12, 13, 14) cited in the guidance from SIGN (3). Studies reported that the younger a child is when they start toothbrushing the lower the proportion developing tooth decay. Data from a cross sectional study of seven year old children showed an increase in the odds of caries when age at commencement of brushing increases by one year (Odds Ratio 1.22; 95% CI 1.14 to 1.30; p<0.001) (13). The evidence is considered very low certainty. The recommendation that “Children should be assisted to brush their teeth as soon as they erupt” is classed as best practice.  There is no high-quality evidence to determine when a parent or guardian should stop assisting/supervising a child’s toothbrushing. Guidelines provide consistent recommendations that teeth should be brushed by a parent or guardian initially and, as the child gets older, the parent or guardian should assist them to brush their own teeth. Supervision should continue until a child is able to brush their own teeth effectively; there is no clear age at which assistance/supervision should stop (1, 3, 8, 9, 11). | |
| **2. Quality and quantity of evidence**  There is no high certainty evidence regarding the optimal age for commencing toothbrushing.  There is no high certainty evidence regarding the age at which supervision/assistance with toothbrushing should stop.  Recommendations are considered ‘best practice’. | |
| 1. **Subgroup considerations**   *-* | |
| **4. Consistency**  Current clinical guidelines are reasonably consistent with regard to recommendations made. | |
| **5. Balance of effects**  *-* | |
| **6. Generalisability and applicability**  There are no major concerns regarding the generalisability or applicability of the recommendations. | |
| **7. Values and preferences**  *-* | |
| **8. Acceptability**  *-* | |
| **9. Feasibility**  *-* | |
| **10. Other factors**  *-* | |

| CJF3 | Considered Judgement for Recommendations |
| --- | --- |
| **Overarching Question**  **Should you rinse or spit before/after brushing?** | |
| **1. Summary of evidence** There are no systematic reviews addressing this question.  Current guidelines recommend that children and adults should be encouraged to spit out excess toothpaste and not rinse with water after brushing. Where guidelines provide underpinning evidence, this has been assessed as low certainty evidence due to the issues around applicability. Two clinical trials are referenced in support of ‘spit don’t rinse’(15, 16). Neither study directly evaluates the benefit of spitting after toothbrushing compared with rinsing. Sjogren et al (1995) evaluated a modified toothpaste technique which included paste to be spread on the teeth prior to brushing alongside advice to not rinse with water after brushing (16). Whilst the modified technique resulted in an average of 26% fewer new proximal carious lesions compared to children in the control groups, the impact of rinsing or spitting alone could not be determined. The trial by Chestnutt et al. (1998) evaluated the impact of different rinsing behaviours, embedded within a clinical trial (15). Children were requested to report how they rinsed their mouth after brushing: “by using a toothbrush to transfer water to the mouth; by putting the mouth under the running tap; by transferring water using cupped hands; or by using a beaker.” Results showed that those who self-reported using a beaker to rinse with water following brushing had a higher caries increment (6.84) compared to those who reported not using a beaker (5.84). | |
| **2. Quality and quantity of evidence**  There is no high certainty evidence regarding whether it is beneficial to rinse or spit following toothbrushing. | |
| **3. Subgroup considerations**   - Provide advice for those who may not be able to spit? - Provide advice on rinsing to dislodge food debris where manual dexterity is limited? - Provide advice regarding supervising very young children to ensure that they spit out excess toothpaste? | |
| **4. Consistency**  - | |
| **5. Balance of effects**  *-* | |
| **6. Generalisability and applicability**  *-* | |
| **7. Values and preferences**  *-* | |
| **8. Acceptability**  *-* | |
| **9. Feasibility**  *-* | |
| **10. Other factors**  *-* | |

| CJF4 | Considered Judgement for Recommendations |
| --- | --- |
| **Toothbrush type**   - **What is the most effective type of toothbrush for maintaining oral health (*powered vs manual)*** - **How effective are miswak/wooden sticks?** - **What is the best toothbrush head type/shape?** | |
| **1. Summary of evidence**  General population  There are multiple systematic reviews that compare powered and manual toothbrushes and different types of powered toothbrushes. Some reviews focus on single-brushing exercises and have been excluded from this assessment as not reflecting daily use.  Reviews comparing powered brushes with manual brushes, whilst showing some variation in inclusion criteria and number of trials included, consistently show a reduction in plaque and gingivitis associated with the use of powered toothbrushes (Table 1 and Table 2). Significant heterogeneity was noted for all analyses.  *Table 1. Powered versus manual toothbrushes: Plaque score*   \| Systematic review \| Time point \| SMD (95% CI) \| I^2^ \| No. of trials (participants) \| \| --- \| --- \| --- \| --- \| --- \| \| de Jager et al. (17) \| 1-3 months \| SMD -0.89 (95% CI -1.27 to -0.51) \| 93% \| 17 (1830) \| \| Yaacob et al. (18) \| 1-3 months \| SMD -0.50 (95% CI -0.70 to -0.31) \| 83% \| 40 (2871) \| \| Yaacob et al. (18) \| > 3 months \| SMD -0.47 (95% CI -0.82 to -0.11); I^2^ = 86%; 14 trials, 978 participants \| 86% \| 14 (978) \| \| Wang et al. (19) \| 1-8 months \| (SMD -0.86 (95% CI: -0.58 to -1.14) \| 91.5% \| 21 \|   *Table 2. Powered versus manual toothbrushes: Gingivitis score*   \| Systematic review \| Time point \| SMD (95% CI) \| I^2^ \| No. of trials (participants) \| \| --- \| --- \| --- \| --- \| --- \| \| de Jager et al. (17) \| 1-3 months \| SMD -0.67 (95% CI -1.01 to -0.32) \| 91% \| 17 (1688) \| \| Yaacob et al. (18) \| 1-3 months \| SMD -0.43 (95% CI -0.60 to -0.25) \| 82% \| 44 (3345) \| \| Yaacob et al. (18) \| > 3 months \| SMD -0.21 (95% CI -0.31 to -0.12) \| 51% \| 16 (1645) \| \| Wang et al. (19) \| 1-8 months \| (SMD -0.47 (95% CI: -0.12 to -0.82) \| 88.7% \| 14 \|   One review, comprising the largest number of trials, presented results as a percentage reduction of the manual toothbrush group. Short-term data showed plaque reductions of approximately 11% and gingivitis reductions of 21%. These reductions were approximately 6% and 11% for studies reporting plaque and gingivitis post-3 months respectively (18).  One systematic review also presented data on decreased bleeding index, showing a benefit in favour of powered brushes (SMD 0.92, 95% CI: 0.43 to 1.40, I2 = 1.8%, p < .0001) (11 trials) (19).  Safety: although not all trials report on safety associated with powered versus manual toothbrushes, one systematic review reports that studies “consistently showed oscillating-rotating toothbrushes to be safe compared to manual toothbrushes, and collectively indicated that they do not pose a clinically relevant concern to either hard or soft tissues.” (20)  Overall, there is moderate-certainty evidence to suggest that powered toothbrushes reduce plaque and gingivitis more than manual toothbrushing in both the short (< 3 months) and long term (> 3 months). The clinical importance of the differences observed is unclear. There are no data regarding caries.  Reviews comparing different types of powered toothbrushes powered brushes address slightly different questions but provide no high certainty evidence of a benefit of one type of powered toothbrush over another. Clark-Perry et al. (2020) report low or moderate certainty evidence that oscillating-rotational powered toothbrushes might remove more plaque and reduce the number of bleedings sites than other powered toothbrushes (namely sonic action toothbrushes). However, the clinical importance of findings is unclear. No difference was shown in terms of the modified gingival index or gingival bleeding index (21).  El-Chami et al. (2021) found no clinically important differences between oscillating-rotational powered toothbrushes and side-to-side powered brushes at any time point. They concluded that both brushes were safe to use (22).  Similarly, Deacon et al. (2010) concluded that no mode of action was consistently superior across all outcomes and time periods studied. There was moderate-certainty evidence to suggest powered oscillating-rotational brushes reduce plaque and gingivitis more than powered side-to-side brushes in the short term, but the difference is small and may not be clinically significant (23).  There is insufficient evidence to determine the effectiveness of different types of wooden sticks/chewing sticks. The benefits/harms associated with the use of these products cannot be determined from the available evidence.  Regarding toothbrush head type, one systematic review suggested that better results for plaque and gingivitis may be achieved from medium or hard toothbrushes (24). However, there was low certainty evidence from a second review that soft and extra-soft toothbrushes tend to be safer, with fewer gingival lesions (25). There is insufficient evidence regarding size of toothbrush head.  Regarding bristle types, there is contradictory evidence. One systematic review concluded that there is insufficient evidence to determine the clinical benefit of manual toothbrush with tapered toothbrush filaments over manual toothbrush with end-rounded toothbrush filaments (26). However, a further review provided very low certainty evidence that, for interproximal surfaces, better results may be expected for tapered-tip bristle toothbrushes when compared to end-rounded bristles toothbrushes (24). There is no evidence of a difference in oral soft tissue injuries between tapered and end-rounded bristles (25).  Specific populations  One review evaluated the effectiveness of powered brushes for maintaining oral hygiene in people with learning difficulties(27). There is low/moderate certainty evidence of no benefit of powered toothbrushes in comparison to manual brushes. The findings were supported by a further systematic review that included limited evidence comparing powered versus manual toothbrushes used by children and adolescents with intellectual disabilities (aged below 18 years old) (28). There is limited evidence to suggest the use of a triple-headed manual toothbrush instead of a single-headed manual toothbrush with respect to plaque removal for care-dependent individuals. However, this is based on ‘vote-counting’ of study results with no indication of the magnitude of effect that might be seen (29). | |
| **2. Quality and quantity of evidence**  Powered/manual toothbrushes:  General population: moderate-certainty evidence.  Adults/children with intellectual difficulties: low/moderate certainty evidence.  *Miswak/wooden sticks*  Very low certainty evidence*.* | |
| **3. Subgroup considerations**   - Need to consider patient’s preferences, SES, manual dexterity etc…. - Consider variations in use by children and adults with physical disabilities who may find a powered toothbrush easier to use - Patients with bridges or dental implants | |
| **4. Consistency**  *Evidence is fairly consistent across reviews with regard to powered/manual toothbrushes* | |
| **5. Balance of effects** Adverse events are rarely reported, however, where they are reported they are minimal and do not appear to outweigh benefits from either powered or manual toothbrushes.  The benefits/harms associated with the use of these products cannot be determined from the available evidence. | |
| **6. Generalisability and applicability**  *No major concerns regarding the generalisability or applicability of the findings regarding powered/manual toothbrushes.* | |
| **7. Values and preferences**  *No evidence regarding preferences in identified reviews* | |
| **8. Acceptability**  *Both powered and manual likely to be acceptable. Possible concerns re use of powered for some vulnerable groups.*  *Costs* | |
| **9. Feasibility**  *Main concern is likely to be differences in cost across the different toothbrushes*  *Access to different types of brushes (inequalities)* | |
| **10. Other factors** *Sustainability?*  *Technique – compliance*  *Replacement of heads/manual brushes* | |

| CJF5 | Considered Judgement for Recommendations |
| --- | --- |
| **Overarching Question**  **How effective are interdental cleaning devices for preventing** **and controlling periodontal diseases and preventing caries?** | |
| **1. Summary of evidence**  There are numerous systematic reviews of interdental brushing and flossing, of varying quality. All are based on trials of unclear or high risk of bias and focus on the reporting of plaque and/or gingivitis.  There is low certainty evidence that interdental cleaning devices, as adjuncts to toothbrushing, removes more dental plaque and reduces gingival inflammation than brushing alone (30, 31, 32, 33). It should be noted however, across comparisons outcomes were typically measured in the short term and participants in most studies had low levels of baseline gingival inflammation. In addition, the clinical importance of the findings remains unclear.  The most comprehensive systematic review reported that there is no evidence to determine whether dental cleaning aids reduce caries when compared to toothbrushing alone (33).  In terms of which interdental cleaning device is the most effective, three reviews reported a potential benefit of interdental brushes as an alternative to flossing in people with gingival inflammation (34), or in the general population (32, 33). The certainty of the evidence is low or very low.  A Bayesian network-meta-analysis determined that interdental brushes and waterjets ranked high for reducing gingival bleeding, while toothpicks and floss ranked lowest. In a follow-on publication that undertook a multi-outcome Bayesian network meta-analysis, with equal weight on gingival inflammation and bleeding on probing, interdental brushes and waterjets remained as the two highest ranking devices across different sets of weightings for gingival inflammation and bleeding on probing (30, 31). The NMA was limited by the small number of studies for certain comparisons and the substantial heterogeneity.  One review focused on the effectiveness of flossing in young adolescents. There was also no evidence of benefit of self-flossing, potentially due to the presence of fluorides, poor flossing techniques, or other reasons (low certainty evidence) (35).  One systematic review evaluated the effectiveness of brushing before flossing compared with flossing before brushing. There was low certainty evidence, from a limited number of studies, of no difference between flossing before or after brushing with regard to a reduction in dental plaque index (36). | |
| **2. Quality and quantity of evidence**  *Evidence is consistently low or very low certainty* | |
| **3. Subgroup considerations**   - *Need to consider patient’s preferences, manual dexterity etc….* - *Children, adults, dependent individuals* - *More important for those at risk of/with, gingivitis or periodontitis* | |
| **4. Consistency**  *Heterogeneity is an issue across some comparisons (and reflected in GRADE assessments). Findings from the systematic reviews are fairly consistent* | |
| **5. Balance of effects**  *Adverse events are rarely reported, however, where they are reported they are minimal and do not outweigh benefits.*  *British Society of Periodontology and Implant Dentistry S3 treatment guidelines states that there is a moderate risk of trauma if interdental brushes are not used correctly but concludes that the benefits of their use far outweigh the risks. It also notes that it is crucial for dental professionals to provide individual instruction in the correct use of interdental brushes.* | |
| **6. Generalisability and applicability**  **-** | |
| **7. Values and preferences**  *No data on preferences*  *The BSP-S3 guideline notes that there is clinical evidence that patients with open interdental spaces prefer the use of interdental brushes to the use of dental floss. It states that patient preferences need to be taken into consideration when discussing the best ways to clean interdentally* | |
| **8. Acceptability**   - *Likely to be acceptable to most patients.* - *Cost* - *The additional time and skill required to clean interdentally may prevent use* | |
| **9. Feasibility**   - *Choice of interdental cleaning approach may depend on spacing of teeth, manual dexterity…* - *Patients may require instruction on how to use interdental cleaning aids effectively.* | |
| **10. Other factors**  Discuss use with GDP or provider | |

| CJF6 | Considered Judgement for Recommendations |
| --- | --- |
| **Overarching question**  **What is the most effective type of toothpaste for maintaining oral health?** | |
| **1. Summary of evidence**  **FLUORIDE TOOTHPASTE**  **What concentration of fluoride toothpaste is most effective?**  There is moderate or high-certainty evidence that fluoride toothpaste of 1000ppm F or above prevents caries in both the permanent and primary dentition (37).  For primary tooth surfaces, three studies evaluated 1500 ppm F compared with 0 ppm F, 1450 ppm F compared with 250 ppm F, and 1055 to 1100 ppm F compared with 500 to 550 ppm F. The greatest caries preventive effect was observed for 1500 ppm F compared with 0 ppm F, where the mean difference in caries surface increment in the higher fluoride group was on average 1.86 lower (95% CI 2.51 to 1.21).  For permanent tooth surfaces in children and adolescents:   - 1000 to 1250 ppm or 1450 to 1500 ppm F toothpaste confers a clinically meaningful caries‐preventive benefit (D(M)FS) when compared with non‐fluoride toothpaste: SMD ‐0.28 (95% CI ‐0.32 to ‐0.25) and ‐0.36 (95% CI ‐0.43 to ‐0.29) - 1450 to 1500 ppm F toothpaste slightly reduces caries increments (D(M)FS) when compared to 1000 to 1250 ppm: SMD -0.08 (-0.14 to -0.01) - No additional caries preventive benefits were observed with higher fluoride concentration toothpaste (1700 to 2200 ppm F and 2400 to 2800 ppm F) toothpaste when compared to 1450 to 1500 ppm F: SMD 0.04 (95% CI -0.07 to 0.15) and SMD -0.05 (95% CI -0.14 to 0.05) - Dose-response analysis shows that approximately 1100 ppm F is needed when compared to non-fluoride toothpaste to reach an SMD of 0.30 (which approximates a prevented fraction of 30%)   For permanent tooth surfaces in adults toothbrushing with 1000 or 1100 ppm F toothpaste reduces DMFS when compared with non‐fluoride toothpaste in adults of all ages: The mean difference in caries surface increment in the higher fluoride group was on average 0.53 lower (95% CI 1.02 to 0.04). The magnitude of this benefit is uncertain as there was considerable variability of effect across studies.  Two systematic reviews evaluating the effect of fluoride toothpastes in orthodontic patients provided low certainty evidence of a benefit of high fluoride toothpaste. Benson et al (2019) concluded that there is low certainty evidence that the use of a high fluoride toothpaste (5000 ppm F) throughout orthodontic treatment, might be more effective than a conventional fluoride toothpaste preventing early tooth decay (demineralised lesions) (38). These results are supported by a network meta-analysis of remineralizing agents in the prevention and reversal of orthodontically induced white spot lesions was undertaken by Hu et al (2020). The review identified evidence from both mixed and indirect comparisons that high fluoride toothpaste (5000ppm) may be beneficial for the prevention/treatment of orthodontically induced white spot lesions (39). GRADE assessment was not possible from the information provided.  **Is there a difference between fluoride formulations (e.g., stannous fluoride vs SMFP vs NaF) in the caries protective effect of toothpastes and if so, are these clinically significant?**  Clark-Perry et al (2020) evaluate the effectiveness of stannous fluoride toothpastes over fluoride toothpastes without stannous fluoride (40). However, the only evidence is on gingival indices and the clinical importance of the size of effect shown is questionable. In addition, there are inconsistencies in the reporting of findings within the review making the results difficult to interpret.  **What is the optimum amount of fluoride toothpaste to use for daily toothbrushing?**  No evidence from RCTs is available for fluorosis, however there is very low quality evidence from cross-sectional studies (41). Results from a pooled analysis of three studies in children with clefts found no significant association between the amount of fluoride toothpaste (small amount/pea-sized versus large/greater than pea-sized amount) and fluorosis was found (OR 0.92, 95% CI 0.67 to 1.28). The studies did not report on caries severity.  **TRICLOSAN TOOTHPASTES**  A single systematic review identified evidence that triclosan/copolymer-containing fluoride toothpastes are beneficial for plaque, gingivitis, periodontitis (low or moderate certainty) and caries (high certainty) when compared with fluoride toothpastes (42). However, the benefits observed are unlikely to be considered clinically important. There was no evidence of any safety concerns, however the duration of the included studies included was too short to investigate any diseases that may develop over a prolonged period. It should be noted that Triclosan has now been removed from Colgate Total toothpaste.  **XYLITOL TOOTHPASTES**  Again, a single systematic review evaluating the effects of different xylitol-containing products for the prevention of dental caries in children and adults identified low certainty evidence to indicate that xylitol-containing fluoride toothpastes reduce caries when compared with fluoride toothpastes (43) . Over 2.5 to 3 years of use, fluoride toothpaste containing 10% xylitol reduced caries when using the decayed and filled surfaces (DFS) index by a mean difference of -0.28 (95% CI -0.42 to -0.14; 2 studies; 4216 participants; heterogeneity: I^2^ = 82%). The reduction observed may not be considered clinically important. There was no evidence of any safety concerns however the duration of the included studies included was too short to investigate any diseases that may develop over a prolonged period**.**  **HERBAL TOOTHPASTES**  There is low or very low certainty evidence regarding the effectiveness of herbal toothpastes for the reduction of dental plaque and gingivitis. Herbal toothpaste may be as effective as non-herbal toothpaste but there is no evidence that they are superior to fluoride toothpaste with regard to plaque and gingivitis outcomes. The review provided no evidence with regard to the effect of herbal toothpastes on caries. The authors concluded that “The quality of evidence appears to be low/very low to recommend them as a substitute to more conventional OTC oral hygiene products” (44).  **ARGININE TOOTHPASTES**  A systematic review evaluating the effectiveness of arginine-containing dental care products in preventing the development of new caries lesions and the progression of existing lesions reported insufficient evidence in support of a caries-preventive effect for the inclusion of arginine in toothpastes in children (45). No trials were identified comparing arginine to fluoride in adults.  However, Castelo et al (2021), when evaluating the management of root caries in middle-aged and older adults identified two trials of 1.5% arginine toothpaste (with fluoride) compared with fluoride only toothpaste (46). Root surface hardness in participants after 6 months of use ranged from 61.7% to 70.5% for arginine-containing toothpaste (with fluoride) compared to 56.0% to 58.1% for fluoride only toothpaste. There is limited evidence that the daily use of a 1.5% arginine toothpaste (with fluoride) may contribute to root caries arrest in older adults. The authors concluded that daily use of a 1.5% arginine toothpaste could also be suggested as a preventive treatment, although this was not directly evaluated in the included trials. | |
| **2. Quality and quantity of evidence**  Most of the evidence evaluates fluoride toothpaste. The studies are typically well conducted and provide moderate to high certainty evidence that fluoride toothpaste of 1000ppm F or above prevents caries in both the permanent and primary dentition.  There is low certainty evidence that the use of a high fluoride toothpaste (5000 ppm F) throughout orthodontic treatment, might be more effective than a conventional fluoride toothpaste preventing early tooth decay.  There is limited evidence with regard to the effect of different fluoride toothpaste formulations. The evidence is limited by risk of bias in the included studies, indirectness of the evidence, imprecision, and heterogeneity.  There is limited, low certainty evidence from two trials with regard to the addition of xylitol to fluoride toothpaste. Xylitol may reduce caries, but the size of effect is unclear.  There is low or very low certainty evidence from over 20 trials with regard to the effect of herbal toothpastes. The evidence is limited by the risk of bias in the included studies and heterogeneity across findings. There is no evidence that herbal toothpastes are superior to fluoride toothpastes for the prevention of plaque or gingivitis.  There is limited evidence with regard to the addition of arginine to fluoride toothpaste for preventing caries. | |
| **3. Subgroup considerations**   - Children, adults, dependent individuals, those with sensory sensitivities may benefit from SLS-free toothpastes (1) - Fluoridated/non-fluoridated areas - Recommended concentration of fluoride in toothpaste may vary according to age group or caries risk. However, a minimum of 1000ppm should be recommended. | |
| **4. Consistency**  - | |
| **5. Balance of effects**  There is weak, unreliable evidence that starting the use of fluoride toothpaste in children under 12 months of age may be associated with an increased risk of mild fluorosis. The evidence for its use between the age of 12 and 24 months is equivocal (47).  There was limited evidence on other adverse events reported for any of the type of toothpaste evaluated. Studies were not always long enough to establish adverse events and not always recorded.  Where adverse events were reported they were minimal. | |
| **6. Generalisability and applicability**  No major concerns regarding the generalisability or applicability of the findings. | |
| **7. Values and preferences** | |
| **8. Acceptability** Whilst most of the evidence comes from the general population, findings may be extrapolated to different groups. | |
| **9. Feasibility** Use of daily toothpaste is feasible in most situations | |
| **10. Other factors** | |

| CJF7 | Considered Judgement for Recommendations |
| --- | --- |
| **Overarching Question**   - ***How should a toothbrush be stored?*** - ***How often should a toothbrush be replaced? **** | |
| **1. Summary of evidence**  There is no high certainty evidence to determine the optimal methods for storing toothbrushes. Guidelines provide best practice recommendations based on very low certainty evidence. One of the most recently updated guidance documents comes from the American Dental Association which recommends:   - Toothbrushes should not be shared.  Sharing a toothbrush could result in an exchange of bodily fluids and microorganisms between people. Evidence to underpin this statement comes from an *in vitro* assessment of the retention of pathogenic microorganisms by different types of toothbrushes (48, 49). - Rinse the toothbrush thoroughly after each use to remove any remaining paste and debris. The statement is based on a non-systematic review that evaluates toothbrush contamination (50). - Store toothbrushes in an upright position after use and allow them to air dry.  Storing a moist toothbrush in a closed container promotes microbial growth more so than leaving it exposed to the open air.   Whilst there is a systematic review evaluating various decontamination methods for cleaning toothbrushes, the evidence appears to be from single disinfection event studies, and it is not clear whether the reductions in microbial counts would be considered clinically important (51). Best practice/consensus recommendations suggest there is no need to soak toothbrushes in disinfecting solutions or to use other sanitizing methods such as microwaves or ultraviolet devices.  There is no high certainty evidence with regard to the frequency of toothbrush replacement. Indeed, previous evidence-based guidelines state “There is insufficient evidence to determine when a worn toothbrush should be replaced” (8). However, current recommendations appear to be reasonably consistent suggesting that toothbrushes should be replaced “every three to four months or more often if the bristles are visibly matted or frayed”(48). | |
| **2. Quality and quantity of evidence**  There is no high certainty evidence regarding the optimal method for storing toothbrushes or replacing them. | |
| **3. Subgroup considerations** | |
| **4. Consistency**  Current clinical guidelines are reasonably consistent in terms of the recommendations made and based on consensus/best practice | |
| **5. Balance of effects**  *-* | |
| **6. Generalisability and applicability**  There are no major concerns regarding the generalisability or applicability of the recommendations presented in identified guidelines. | |
| **7. Values and preferences**  *-* | |
| **8. Acceptability**  *-* | |
| **9. Feasibility**  *-* | |
| **10. Other factors**  *-* | |

References

1. Department of Health. Delivering better oral health: an evidence-based toolkit for prevention. 2021.

2. Registered Nurses’ Association of Ontario (RNAO). Oral health: Supporting adults who require assistance. 2nd ed. Toronto (ON): RNAO; 2020.

3. Scottish Intercollegiate Guidelines Network (SIGN). Dental interventions to prevent caries in children. Edinburgh: SIGN; 2014. (SIGN publication no. 138). [March 2014]. Available from URL: <http://www.sign.ac.uk>.

4. American Academy of Pediatric Dentistry. Guideline on Perinatal and Infant Oral Health Care. Pediatr Dent. 2016;38(6):150-4.

5. AWMF. Caries prevention in permanent teeth − basic recommendations. AWMF online; 2016.

6. National Institute for Health and Care Excellence. Oral health in care homes. Quality Standard (QS151). 2017.

7. Scottish Dental Clinical Effectiveness Programme. Prevention and Treatment of Periodontal Diseases in Primary Care. SDCEP; 2014.

8. Scottish Dental Clinical Effectiveness Programme. Prevention and Management of Dental Caries in Children. SDCEP; 2018.

9. Working group established by Duodecim of the Finnish Medical Society and the Finnish Dental Society Apollonia. Caries (management). Valid treatment recommendation. Helsinki: Suomalainen Lääkäriseura Duodecim, 2020. Available on the internet: <www.kaypahoito.fi>.

10. Marinho VC, Higgins JP, Sheiham A, Logan S. Fluoride toothpastes for preventing dental caries in children and adolescents. Cochrane Database Syst Rev. 2003;2003(1):CD002278.

11. American Academy of Pediatric Dentistry. Clinical Affairs Committee--Infant Oral Health S. Guideline on infant oral health care. Pediatr Dent. 2012;34(5):e148-52.

12. Hinds K, Gregory J. National diet and nutrition survey: children aged 1.5 to 4.5 years. London: HMSO; 1995.

13. Vanobbergen J, Martens L, Lesaffre E, Bogaerts K, Declerck D. Assessing risk indicators for dental caries in the primary dentition. Community Dent Oral Epidemiol. 2001;29(6):424-34.

14. Verrips G, Kalsbeek H, Van Woerkum C, Koelen M, Kok-Weimar T. Correlates of toothbrushing in preschool children by their parents in four ethnic groups in The Netherlands. Community Dent Health. 1994;11(4):233-9.

15. Chestnutt I, Schäfer F, Jacobson A, Stephen K. The influence of toothbrushing frequency and post-brushing rinsing on caries experience in a caries clinical trial. Community Dentistry Oral Epidemiology. 1998;26(6):406-11.

16. Sjogren K, Birkhed D, Rangmar B. Effect of a modified toothpaste technique on approximal caries in preschool children. Caries Res. 1995;29(6):435-41.

17. de Jager M, Rmaile A, Darch O, Bikker JW. The Effectiveness of Manual versus High-Frequency, High-Amplitude Sonic Powered Toothbrushes for Oral Health: A Meta-Analysis. J Clin Dent. 2017;28(1 Spec No A):A13-28.

18. Yaacob M, Worthington HV, Deacon SA, Deery C, Walmsley AD, Robinson PG, et al. Powered versus manual toothbrushing for oral health. Cochrane Database Syst Rev. 2014;2014(6):CD002281.

19. Wang P, Xu Y, Zhang J, Chen X, Liang W, Liu X, et al. Comparison of the effectiveness between power toothbrushes and manual toothbrushes for oral health: a systematic review and meta-analysis. Acta Odontol Scand. 2020;78(4):265-74.

20. Van der Weijden FA, Campbell SL, Dorfer CE, Gonzalez-Cabezas C, Slot DE. Safety of oscillating-rotating powered brushes compared to manual toothbrushes: a systematic review. J Periodontol. 2011;82(1):5-24.

21. Clark-Perry D, Levin L. Systematic review and meta-analysis of randomized controlled studies comparing oscillating-rotating and other powered toothbrushes. J Am Dent Assoc. 2020;151(4):265-75 e6.

22. El-Chami H, Younis A, Brignardello-Petersen R. Efficacy of oscillating rotating versus side-to-side powered toothbrushes on plaque and gingival index reduction: A systematic review. J Am Dent Assoc. 2021;152(2):115-26 e4.

23. Deacon SA, Glenny AM, Deery C, Robinson PG, Heanue M, Walmsley AD, et al. Different powered toothbrushes for plaque control and gingival health. Cochrane Database Syst Rev. 2010;2010(12):CD004971.

24. Langa GPJ, Muniz F, Wagner TP, Silva CFE, Rosing CK. Anti-Plaque and Anti-Gingivitis Efficacy of Different Bristle Stiffness and End-Shape Toothbrushes on Interproximal Surfaces: A Systematic Review with Meta-Analysis. J Evid Based Dent Pract. 2021;21(2):101548.

25. Ranzan N, Muniz F, Rosing CK. Are bristle stiffness and bristle end-shape related to adverse effects on soft tissues during toothbrushing? A systematic review. Int Dent J. 2019;69(3):171-82.

26. Hoogteijling F, Hennequin-Hoenderdos NL, Van der Weijden GA, Slot DE. The effect of tapered toothbrush filaments compared to end-rounded filaments on dental plaque, gingivitis and gingival abrasion: a systematic review and meta-analysis. Int J Dent Hyg. 2018;16(1):3-12.

27. Waldron C, Nunn J, Mac Giolla Phadraig C, Comiskey C, Guerin S, van Harten MT, et al. Oral hygiene interventions for people with intellectual disabilities. Cochrane Database Syst Rev. 2019;5(5):CD012628.

28. Zhou N, Wong HM, Wen YF, McGrath C. Efficacy of caries and gingivitis prevention strategies among children and adolescents with intellectual disabilities: a systematic review and meta-analysis. J Intellect Disabil Res. 2019;63(6):507-18.

29. Kalf-Scholte SM, Van der Weijden GA, Bakker E, Slot DE. Plaque removal with triple-headed vs single-headed manual toothbrushes-a systematic review. Int J Dent Hyg. 2018;16(1):13-23.

30. Kotsakis GA, Lian Q, Ioannou AL, Michalowicz BS, John MT, Chu H. A network meta-analysis of interproximal oral hygiene methods in the reduction of clinical indices of inflammation. J Periodontol. 2018;89(5):558-70.

31. Liang M, Lian Q, Kotsakis GA, Michalowicz BS, John MT, Chu H. Bayesian Network Meta-analysis of Multiple Outcomes in Dental Research. J Evid Based Dent Pract. 2020;20(1):101403.

32. Slot DE, Dorfer CE, Van der Weijden GA. The efficacy of interdental brushes on plaque and parameters of periodontal inflammation: a systematic review. Int J Dent Hyg. 2008;6(4):253-64.

33. Worthington HV, MacDonald L, Poklepovic Pericic T, Sambunjak D, Johnson TM, Imai P, et al. Home use of interdental cleaning devices, in addition to toothbrushing, for preventing and controlling periodontal diseases and dental caries. Cochrane Database Syst Rev. 2019;4(4):CD012018.

34. Imai P, Yu X, MacDonald D. Comparison of interdental brush to dental floss for reduction of clinical parameters of periodontal disease: a systematic review. Canadian Journal of Dental Hygiene. 2012;46(1):63-78.

35. Hujoel PP, Cunha-Cruz J, Banting DW, Loesche WJ. Dental flossing and interproximal caries: a systematic review. J Dent Res. 2006;85(4):298-305.

36. Silva C, Albuquerque P, de Assis P, Lopes C, Anníbal H, Lago M, et al. Does flossing before or after brushing influence the reduction in the plaque index? A systematic review and meta-analysis. Int J Dent Hyg. 2022;20(1):18-25.

37. Walsh T, Worthington HV, Glenny AM, Marinho VC, Jeroncic A. Fluoride toothpastes of different concentrations for preventing dental caries. Cochrane Database Syst Rev. 2019;3(3):CD007868.

38. Benson PE, Parkin N, Dyer F, Millett DT, Germain P. Fluorides for preventing early tooth decay (demineralised lesions) during fixed brace treatment. Cochrane Database Syst Rev. 2019;2019(11).

39. Hu H, Feng C, Jiang Z, Wang L, Shrestha S, Yan J, et al. Effectiveness of remineralizing agents in the prevention and reversal of orthodontically induced white spot lesions: a systematic review and network meta-analysis. Clin Oral Investig. 2020;24(12):4153-67.

40. Clark-Perry D, Levin L. Comparison of new formulas of stannous fluoride toothpastes with other commercially available fluoridated toothpastes: A systematic review and meta-analysis of randomised controlled trials. Int Dent J. 2020;70(6):418-26.

41. Wong FW, King NM. The oral health of children with clefts--a review. Cleft Palate Craniofac J. 1998;35(3):248-54.

42. Riley P, Lamont T. Triclosan/copolymer containing toothpastes for oral health. Cochrane Database Syst Rev. 2013;2013(12):CD010514.

43. Riley P, Moore D, Ahmed F, Sharif MO, Worthington HV. Xylitol-containing products for preventing dental caries in children and adults. Cochrane Database Syst Rev. 2015;2015(3):CD010743.

44. Janakiram C, Venkitachalam R, Fontelo P, Iafolla TJ, Dye BA. Effectiveness of herbal oral care products in reducing dental plaque & gingivitis - a systematic review and meta-analysis. BMC Complement Med Ther. 2020;20(1):43.

45. Astvaldsdottir A, Naimi-Akbar A, Davidson T, Brolund A, Lintamo L, Attergren Granath A, et al. Arginine and Caries Prevention: A Systematic Review. Caries Res. 2016;50(4):383-93.

46. Castelo R, Attik N, Catirse A, Pradelle-Plasse N, Tirapelli C, Grosgogeat B. Is there a preferable management for root caries in middle-aged and older adults? A systematic review. Br Dent J. 2021.

47. Wong MC, Glenny AM, Tsang BW, Lo EC, Worthington HV, Marinho VC. Topical fluoride as a cause of dental fluorosis in children. Cochrane Database Syst Rev. 2010;2010(1):CD007693.

48. American Dental Association. 2019 [Available from: <http://www.ada.org/en/about-the-ada/ada-positions-policies-and-statements/statement-on-toothbrush-care-cleaning-storage-and-external>.

49. Bunetel L, Tricot-Doleux S, Agnani G, Bonnaure-Mallet M. In vitro evaluation of the retention of three species of pathogenic microorganisms by three different types of toothbrush. Oral Microbiol Immunol. 2000;15(5):313-6.

50. Frazelle M, Munro C. Toothbrush contamination: a review of the literature. Nursing research and practice. 2012;420630.

51. Agrawal SK, Dahal S, Bhumika TV, Nair NS. Evaluating Sanitization of Toothbrushes Using Various Decontamination Methods: A Meta-Analysis. J Nepal Health Res Counc. 2019;16(41):364-71.
